# The Fabrication of a Probe-Integrated Electrochemiluminescence Aptasensor Based on Double-Layered Nanochannel Array with Opposite Charges for the Sensitive Determination of C-Reactive Protein

**DOI:** 10.3390/molecules28237867

**Published:** 2023-11-30

**Authors:** Feng Li, Qianqian Han, Fengna Xi

**Affiliations:** 1 Shanxi Bethune Hospital, Shanxi Academy of Medical Sciences, Tongji Shanxi Hospital, Third Hospital of Shanxi Medical University, Taiyuan 030032, China; lifeng1010@sxmu.edu.cn; 2 Tongji Hospital, Tongji Medical College, Huazhong University of Science and Technology, Wuhan 430030, China; 3 School of Chemistry and Chemical Engineering, Zhejiang Sci-Tech University, Hangzhou 310018, China; 202130107292@mails.zstu.edu.cn

**Keywords:** aptasensor, electrochemiluminescence, bipolar silica nanochannel film, probe-integrated, C-reactive protein

## Abstract

The effective and sensitive detection of the important biomarker, C-reactive protein (CRP), is of great significance in clinical diagnosis. The development of a convenient and highly sensitive electrochemiluminescence (ECL) aptasensor with an immobilized emitter probe is highly desirable. In this work, a probe-integrated ECL aptamer sensor was constructed based on a bipolar silica nanochannel film (bp-SNF) modified electrode for the highly sensitive ECL detection of CRP. The bp-SNF, modified on an ITO electrode, consisted of a dual-layered SNF film, including the negatively charged inner SNF (n-SNF) and the outer SNF with a positive charge and amino groups (p-SNF). The ECL emitter, tris(bipyridine) ruthenium (II) (Ru(bpy)_3_^2+^), was stably immobilized in a nanochannel of bp-SNF using the dual electrostatic interactions with n-SNF attracting and p-SNF repelling. The amino groups on the outer surface of bp-SNF were aldehyde derivatized, allowing for the covalent immobilization of recognitive aptamers (5′-NH_2_-CGAAGGGGATTCGAGGGGTGATTGCGTGCTCCATTTGGTG-3′), leading to the recognition interface. When CRP bound to the aptamer on the recognition interface, the formed complex increased the interface resistance and reduced the diffusion of the co-reactant tripropylamine (TPA) into the nanochannels, leading to a decrease in the ECL signal. Based on this mechanism, the constructed aptamer sensor could achieve a sensitive ECL detection of CRP ranging from 0.01 to 1000 ng/mL, with a detection limit (DL) of 8.5 pg/mL. The method for constructing this probe-integrated ECL aptamer sensor is simple, and it offers a high probe stability, good selectivity, and high sensitivity.

## 1. Introduction

C-reactive protein (CRP) is a protein present in the human blood, and it plays a crucial role in the immune system’s responses [1,2]. As an acute-phase protein synthesized by liver cells, its production levels can rapidly increase in situations such as inflammation or infection [3,4,5]. Thus, the primary physiological function of CRP is to participate in the inflammatory response of the immune system. When the body is exposed to infection, injury, or inflammation, the production of CRP increases rapidly [6,7,8]. It can bind to molecules on the surface of pathogens or damaged cells, activating the immune system and assisting the body in dealing with inflammation and infection. For instance, elevated CRP levels are associated with inflammation and cardiovascular risk in individuals with diabetes [9,10]. As an important biomarker in clinical diagnosis, CRP detection is also used in clinical medicine to detect and monitor various diseases, including infections, autoimmune disorders, and certain tumors [11,12]. Therefore, the effective and sensitive detection of CRP is crucial for a better understanding of a patient’s immune status and overall health.

With the continuous development of molecular biology technologies, biosensors have attracted widespread attention in the determination of CRP in real samples [9]. Current methods for quantifying CRP mainly rely on specific recognition reactions between antibodies and antigens. Compared to antibodies, which are natural immune receptors, aptamers exhibit a high degree of specific binding affinity to target analytes through the formation of two-dimensional or three-dimensional structures [13,14,15]. Aptamers have a wide range of targets, covering various types such as small molecules, proteins, cells, and even tissues [16,17]. In addition, aptamers are single-stranded nucleic acid recognition molecules that typically have molecular sizes smaller than 25 kDa, making them more amenable to chemical synthesis and possessing an excellent stability [18,19]. Common methods for signal detection in aptamer-based sensors include fluorescence, electrochemistry [20,21,22], and electrochemiluminescence (ECL) [23]. ECL technology has gained significant attention due to its high sensitivity, rapid response, simplicity, and portability [24,25,26]. Since CRP is typically a protein without ECL activity, its detection primarily relies on monitoring changes in the ECL signal system during the binding process between aptamers and CRP. Two common detection modes are based on free or immobilized ECL emitters [27,28,29]. The former often requires the addition of free ECL emitters to the test solution, but this may necessitate more reagents and the sample handling steps and is susceptible to sample contamination. In contrast, ECL emitters are immobilized on the electrode surface in the latter mode. There is no need to add ECL probes to the test solution, which is fast, convenient, and requires no complex sample handling. It can achieve online, continuous, and real-time monitoring. Therefore, the development of a convenient and highly sensitive ECL aptamer sensor with immobilized emitters is of significance for CRP determination.

The use of different functional nanomaterials can remarkably enhance the performances of sensors [30,31,32,33]. On one hand, nanomaterials typically possess an extremely high surface area, providing a greater reactive surface area compared to traditional materials [34,35,36]. This increases the contact area with the analyte, improving the sensitivity of the sensor [37,38]. In addition, nanomaterials can be employed as carriers for probe immobilization, ensuring the stability and reusability of the probe. Among these nanomaterials, silica nanochannel film (SNF) with vertically oriented and ultrasmall nanochannels is attractive [39,40,41,42]. In addition to the excellent heat resistance and stability of silica materials, the nanochannel structure of SNF provides an exceptionally high surface area, aiding in the efficiency of adsorption, reactions, and small molecule transport [43,44]. At the same time, the uniform and ultra-small apertures of SNF enable the selective passage of molecules with specific sizes, enabling precise molecular sieving [45,46,47]. For example, SNF can exclude proteins, DNA, suspended particles, and other components from entering the nanochannels, demonstrating a size exclusion effect. Combined with its non-conductive silica structure, an SNF modifier can protect the electrode from contamination, reducing background interference and enabling the direct electrochemical detection of complex biological samples such as serum [48,49,50,51]. Additionally, the silanol groups (Si-OH) on the surface of SNF have a low p*K*_a_ (~2), rendering them a negatively charged surface in typical solution media, facilitating molecular separation and detection [39]. For instance, SNF can significantly electrostatically enrich positively charged small molecules while electrostatically repelling negatively charged small molecules [52]. Functional group modification by introducing positively charged groups can achieve charge reversal and alter charge selectivity permeability [53,54]. Therefore, SNF-modified electrodes hold tremendous potential in the construction of high-performance ECL aptamer sensors.

**Figure 1:**
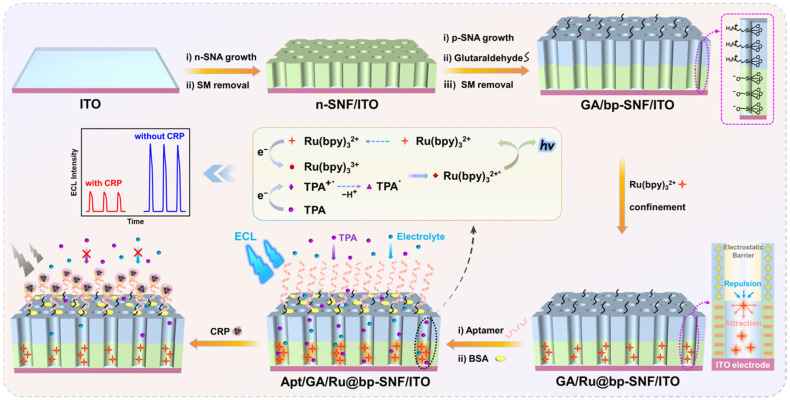
Schematic illustration for the fabrication of the ECL emitter-immobilized aptasensor based on bp-SNF-modified ITO electrode for ECL determination of CRP. For simplicity in illustration, the glass substrate of the ITO electrode was not illustrated and only the ITO layer was schematically represented.

In this work, the sensitive detection of CRP was achieved by constructing a probe-immobilized ECL aptamer sensor based on a bipolar SNF (bp-SNF) with different charge properties. As shown in Figure 1, an inexpensive and readily available indium tin oxide (ITO) electrode was used as the supporting electrode. Initially, a negatively charged SNF (n-SNF) was grown on the electrode surface, followed by the introduction of amino groups to grow positively charged SNF (p-SNF) on top, resulting in bp-SNF-modified electrodes (bp-SNF/ITO). The commonly used ECL emitter, tris(bipyridine)ruthenium (Ru(bpy)_3_^2+^), was confined by the electrostatic interactions within the bp-SNF, resulting in a Ru@bp-SNF/ITO electrode. The high-density nanochannels enabled the loading of a large quantity of Ru(bpy)_3_^2+^ within the nanospace, enhancing the ECL signal. The dual electrostatic forces within each layer of bp-SNF stably confined Ru(bpy)_3_^2+^ at the n-SNF nanochannels, preventing probe leakage and enhancing the stability of the immobilized probes. Consequently, the confined Ru(bpy)_3_^2+^ serves as an efficient solid-state ECL emitter, generating a strong and stable ECL signal. In addition, the amino groups on the external surface of bp-SNF were derivatized with aldehyde groups, allowing for the covalent immobilization of amino-modified aptamers. After blocking nonspecific sites, an aptamer recognition interface was constructed. The binding of CRP to the aptamer on the recognition interface resulted in an increased interface resistance and reduced diffusion of co-reactant to the underlying electrode simultaneously, causing a decrease in the ECL signal. The ECL aptamer sensor developed in this study offers advantages such as a high sensitivity and stability, making it advantageous for sensitive biomarker detection.

## 2. Results and Discussion

### 2.1. Construction of a ECL Probe-Integrated Aptasensor

The key for the construction of a probe-immobilized ECL aptamer sensor lies in the effective immobilization of both an ECL emitter and recognitive aptamer. As depicted in Figure 1, two layers of SNF with asymmetric surface charges were sequentially grown on the ITO electrode surface. These two layers of SNF were grown using different precursor solutions, resulting in distinct functional groups and surface charge properties. Firstly, negatively charged SNF (n-SNF) was grown on the ITO electrode using the Stöber solution growth method, leading to n-SNF/ITO with surfactant micelles (SM), named SM@n-SNF/ITO. The Stöber solution growth method allows for the one-step growth of a large area of n-SNF. After removing the SM from the silica nanochannels, the resulting n-SNF surface carried negative charges due to the deprotonation of silanol groups. Subsequently, the electrode with n-SNF/ITO was subjected to an electrochemical-assisted self-assembly (EASA) method in a precursor solution containing amino-functionalized siloxane. This process led to the growth of positively charged p-SNF with abundant amino groups. EASA is a rapid method for growing SNFs on an electrode surface, where the mechanism involves the electrochemically controlled sol–gel process of siloxane precursors under the template of surfactant micelles, allowing for the growth of SNF on the electrode surface within 10 s.

After the growth of p-SNF, the nanochannels within bp-SNF contained surfactant template micelles. Amino groups on the outer surface of bp-SNF were then derivatized using aldehyde groups. Since the SM blocked the nanochannels of p-SNF, aldehyde derivatization only occurred on the outer surface of bp-SNF, avoiding the cross-linking of amino groups inside the nanochannels. Subsequently, after removing the micelles, the obtained GA/bp-SNF/ITO electrode was stirred in a solution containing Ru(bpy)_3_^2+^. Ru(bpy)_3_^2+^ entered the interior n-SNF under the action of the stirring force and was electrostatically captured to obtain the GA/Ru@bp-SNF/ITO electrode. After the covalent immobilization of the aptamer on the aldehyde-functionalized outer surface, non-specific sites were blocked using bovine serum albumin (BSA) to construct the adapter bio-recognition interface. When CRP specifically recognized the adapter, the interface resistance increased due to the non-conductivity of the formed complex. Coupled with its larger size, the diffusion of the co-reactant TPA was hindered, resulting in a decrease in the ECL signal of Ru(bpy)_3_^2+^. Based on this mechanism, the sensitive ECL detection of CRP could be achieved. The fabricated aptasensor benefited from an inexpensive and readily available supporting electrode, while the convenient and low-cost fabrication of bp-SNF resulted in a significant immobilization effect on the ECL probe and recognitive bio-ligands. The constructed probe-immobilized adapter sensing platform holds promise for the rapid and highly sensitive detection of CRP.

### 2.2. Characterization of the Morphology and Charge Characteristics of bp-SNF

**Figure 2:**
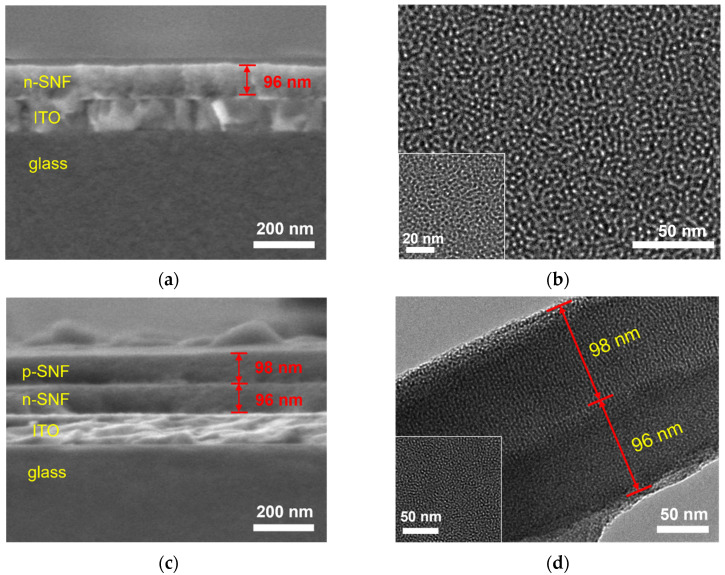
(**a**) Cross-sectional SEM image of n-SNF/ITO. (**b**) Top-view TEM image of n-SNF. Inset was the high-resolution TEM image. (**c**) Cross-sectional SEM image of bp-SNF/ITO. (**d**) TEM image of cross-section of bp-SNF. Inset was the top-view TEM image of p-SNF.

The morphology of n-SNF and bp-SNF and the corresponding electrode were characterized using scanning electron microscopy (SEM) and transmission electron microscopy (TEM). In Figure 2a, cross-sectional SEM image of the n-SNF/ITO electrode was present. It is evident that the ITO layer on the glass substrate formed the ITO electrode. Further growth of n-SNF revealed a three-layer structure. As shown, n-SNF uniformly grew on the ITO surface, displaying a smooth surface with a thickness of 96 nanometers. Figure 2b shows a top-view TEM image of the n-SNF surface. The nanochannels in n-SNF exhibited a continuous structure without any fracture, and their diameter fell within the range from 2 to 3 nanometers. Figure 2c displays a cross-sectional SEM image of the bp-SNF/ITO electrode, where four distinct layers can be clearly observed from top to bottom, corresponding to p-SNF (with a thickness of 98 nanometers), n-SNF (with a thickness of 96 nanometers), ITO, and the glass substrate. Figure 2c presents a TEM image of the cross-section of bp-SNF. It reveals that bp-SNF consisted of vertically oriented double layers of SNF, with nanochannel lengths measuring approximately 98 nanometers and 96 nanometers. The inset TEM image in Figure 2d provides a top-view image of bp-SNF, showing that the nanochannels were arranged continuously, with pore diameters ranging from 2 to 3 nanometers.

**Figure 3:**
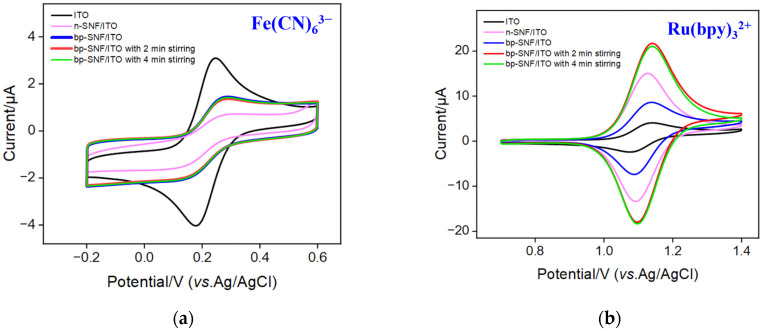
(**a**) CV curves obtained on ITO, n-SNF/ITO, and bp-SNF/ITO electrodes without or with 2 or 4 min stirring in KHP (0.05 M, pH = 4) containing 50 μM Fe(CN)_6_^3−^. (**b**) CV curves obtained on ITO, n-SNF/ITO, and bp-SNF/ITO without or with 1, 2, or 4 min stirring in KHP (0.05 M, pH = 4) containing 50 μM Ru(bpy)_3_^2+^. The scan rate was 50 mV/s.

Due to the different siloxane precursors used in the growth of SNF, bp-SNF exhibited asymmetric surface charges, consisting of an inner layer of negatively charged n-SNF and an outer layer of positively charged p-SNF. By investigating the cyclic voltammetry (CV) curves of two electrochemical probes with different charges, including the anionic probe Fe(CN)_6_^3−^ and the cationic probe Ru(bpy)_3_^2+^, on different electrodes, the charge-selective permeability of the SNF film was studied in potassium hydrogen phthalate (KHP, pH 4.0) electrolyte medium [45,46,47]. Figure 3a presents the CV curves obtained on the ITO, n-SNF/ITO, and bp-SNF/ITO electrodes in the presence of the negatively charged Fe(CN)_6_^3−^ probe. Compared to bare ITO, the n-SNF-modified ITO electrode (n-SNF/ITO) exhibited significantly reduced oxidation or reduction peaks of the Fe(CN)_6_^3−^ probe. This decrease in peak current can be attributed to electrostatic repulsion from n-SNF. Specifically, the abundant silanol groups (Si-OH) with a low p*K*_a_ (~2) ionized in the testing buffer, resulting in a negatively charged surface and corresponding electrostatic repulsion towards anionic Fe(CN)_6_^3−^. When p-SNF was further grown to obtain bp-SNF/ITO, the peak peaks of the Fe(CN)_6_^3−^ slightly increased. This was because the p-SNF surface carried amino groups with positive charges, allowing for the electrostatic adsorption of Fe(CN)_6_^3−^. Continuous stirring maintained a consistent current signal for bp-SNF/ITO in the negatively charged Fe(CN)_6_^3−^. However, the situation was significantly different when testing in a solution of the positively charged Ru(bpy)_3_^2+^ probe. As shown in Figure 3b, compared to bare ITO, the n-SNF/ITO electrode displayed a significantly higher peak current of Ru(bpy)_3_^2+^, resulting from the electrostatic adsorption by n-SNF. When the second p-SNF layer was grown to obtain bp-SNF/ITO, the signal of the Ru(bpy)_3_^2+^ probe decreased compared to that on the n-SNF/ITO electrode because of the electrostatic repulsion from p-SNF. Consequently, when both the negatively charged Fe(CN)_6_^3−^ or positively charged Ru(bpy)_3_^2+^ probes passed through the nanochannels of the bp-SNF/ITO electrode, they were affected by synergistic electrostatic effects from both the outer p-SNF layer and the inner n-SNF layer. When the electrode was immersed in a stirred solution of Ru(bpy)_3_^2+^ for 2 min, a significant increase in peak current was observed. After stirring for 4 min, the electrode signal was nearly identical to that after 2 min of stirring. This suggests that stirring facilitated Ru(bpy)_3_^2+^ to overcome the electrostatic repulsion of the outer layer, enabling the rapid enrichment of Ru(bpy)_3_^2+^ within the inner SNF.

### 2.3. Stable Confinement of Ru(bpy)_3_^2+^ by bp-SNF/ITO Electrode

**Figure 4:**
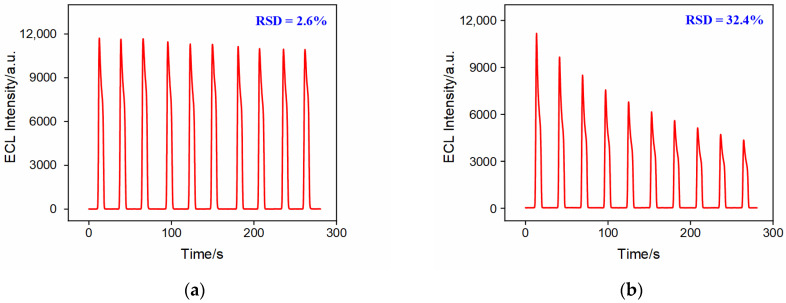
Time-dependent ECL signals during continuous CV scans obtained from Ru@bp-SNF/ITO (**a**) and Ru@n-SNF/ITO (**b**) electrodes in 0.01 M PBS (pH 7.4) containing 3 mM TPA, PMT = 400 V. In CV scan, the scan rate was 100 mV/s and the scanned potential ranged from 0 V to 1.4 V. Time for one ECL signal measurement was 28 s.

The stability of the ECL emitter immobilized on the bp-SNF/ITO electrode was investigated by measuring the ECL signal on the Ru@bp-SNF/ITO electrode during continuous scans. For comparison, the ECL signal on the Ru@n-SNF/ITO electrode was also studied as a control. As shown in Figure 4a, the ECL signal of the Ru@bp-SNF/ITO electrode in a solution containing co-reactants remained highly stable, with a relative standard deviation (RSD) of 2.6% for the ECL intensity during ten consecutive scans. This indicated that Ru(bpy)_3_^2+^ confined within bp-SNF exhibited a remarkable stability. In contrast, the RSD for the ECL intensity obtained from ten consecutive CV scans of the Ru@n-SNF/ITO electrode was 32.4% (Figure 4b). In addition, the ECL intensity retained only 38.9% of its initial signal after ten scans. This suggested that Ru(bpy)_3_^2+^ confined within n-SNF inevitably diffused into the electrolyte solution due to concentration polarization, resulting in a lower stability. It is worth noting that a slightly lower ECL signal was obtained on the Ru@n-SNF/ITO electrode compared to that of the Ru@bp-SNF/ITO electrode. As stirring facilitated Ru(bpy)_3_^2+^ to overcome the electrostatic repulsion of the outer layer to enter the inner n-SNF, the fixed amount of probe depended on the adsorption capacity of n-SNA. Thus, the probe enriched on the Ru@n-SNF/ITO and Ru@bp-SNF/ITO electrodes should be similar. In the preparation process, the SNF/ITO or bp-SNF/ITO electrode was immersed in a stirred Ru(bpy)_3_^2+^ solution, washed with ultrapure water, and then measured. During this process, some probes from n-SNA might have diffused into the ultrapure water used for cleaning due to concentration polarization, resulting in a slightly lower signal compared to that of the Ru@bp-SNF/ITO electrode. The high stability of Ru(bpy)_3_^2+^ confined within bp-SNF can be attributed to the dual electrostatic interaction forming the two-layer SNF of bp-SNF. As depicted in Figure 1, the confined Ru(bpy)_3_^2+^ experienced both electrostatic repulsion from the outer p-SNF and electrostatic attraction from the inner n-SNF. This dual electrostatic interaction effectively prevented probe leakage. Therefore, bp-SNF with asymmetric surface charges could act asa electrostatic nanocage array to stably confine Ru(bpy)_3_^2+^, achieving the stable immobilization of the ECL emitter on the electrode surface.

### 2.4. Feasibility of Constructing ECL Aptasensor

**Figure 5:**
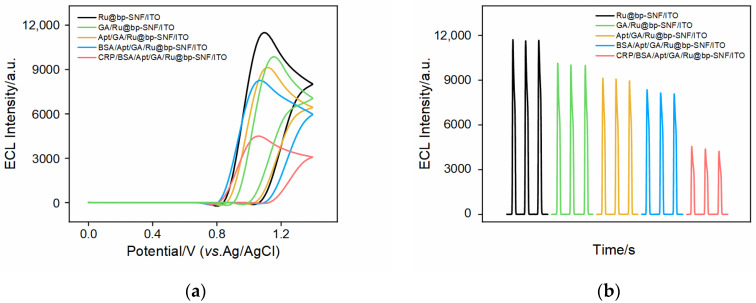
(**a**) ECL-potential curves and (**b**) ECL-time curves obtained in different electrodes in 0.01 M PBS (pH 7.4) containing 3 mM TPA, PMT = 400 V.

Using TPA as the co-reactant for the immobilized Ru(bpy)_3_^2+^, the feasibility for the construction of ECL aptasensors was validated by examining the ECL-potential curves (Figure 5a) and ECL-time curves (Figure 5b) of the different electrodes obtained during the sensor construction process. It can be observed that, as GA cross-linking and aptamer immobilization occurred, the ECL signal gradually decreased. After blocking non-specific sites using BSA, the ECL signal further decreased. As known, BSA (3v03 of Proteindata Bank-PDB) is a globular protein with a molecular weight of 66.5 kDa, comprising 607 amino acids and is ~10 nm in size (pH > 4.7). Due to the ultra-small nanochannels of SNA, BSA was unable to enter these nanochannels. However, when bound to the outer surface of bp-SNA to block non-specific sites, its large protein structure increased the interface resistance and reduced the diffusion of ECL co-reactants to the underlying electrode, resulting in a decrease in the ECL signal. When the constructed ligand sensor was incubated with CRP, the ECL signal of the electrode further decreased. This was due to the specific binding of the aptamer to CRP. The formed aptamer–antigen complex further reduced the diffusion of co-reactant, confirming the successful construction of the aptasensor.

### 2.5. Optimization of the Fabrication of the Aptasensor

**Figure 6:**
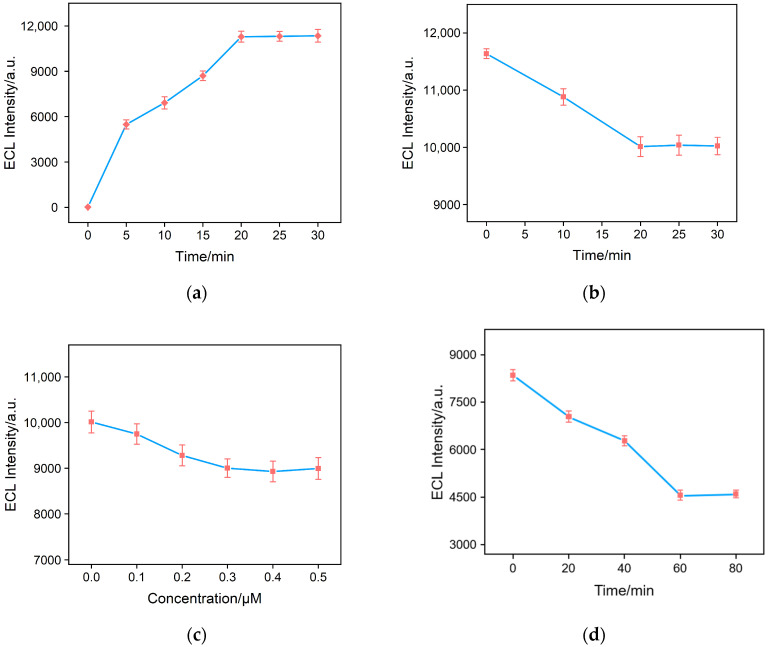
(**a**) Time-dependent ECL signals obtained on bp-SNF/ITO electrode after stirring in 1 mM Ru(bpy)_3_^2+^ for different time. (**b**) The ECL signals obtained when the aptasensor fabricated with different GA reaction time with SM@bp-SNF/ITO for CRP detection. (**c**) The ECL signals obtained on GA/Ru@bp-SNF/ITO electrode fabricated using different concentrations of incubated Apt in 0.01 M PBS (pH 7.4) containing 3 mM TPA, PMT = 400 V. (**d**) ECL signals obtained when the BSA/Apt/GA/Ru@bp-SNF/ITO electrode incubated with 10 ng/mL CRP for different times.

To achieve tje optimal detection performance, the process for the fabrication of the aptasensor was optimized. including the stirring time for Ru(bpy)_3_^2+^ enrichment, the GA reaction time for surface aldehyde derivatization, the concentration of the aptamer in constructing the sensing interface, and the incubation time for CRP detection. Figure 6a shows the ECL signals of the electrodes obtained by immersing GA/bp-SNF/ITO electrodes in a Ru(bpy)_3_^2+^ solution with different stirring times. It can be observed that, with increasing the stirring time, the ECL intensity gradually increased and stabilized. Thus, 20 min of enrichment was chosen for subsequent studies. Before removing the micelles from the SM@bp-SNF/ITO electrode, GA derivatization was performed, allowing GA to be selectively modified on the outer surface of p-SNF without blocking the channels. Figure 6b shows the change in the ECL intensity when detecting CRP using sensors fabricated with different GA reaction times. The optimal reaction time for GA derivatization was 20 min. In the construction of the sensing interface, the NH_2_-modified aptamer was covalently immobilized on the aldehyde-functionalized outer surface of bp-SNF. Different concentrations of aptamers were incubated at 4 °C for 1 h, and the ECL intensity when detecting CRP using the obtained sensors is shown in Figure 6c. The optimal ligand concentration was found to be 0.3 μM. After incubating the prepared aptasensor with 10 ng/mL CRP at 4 °C for different times, the ECL intensity of the electrode is shown in Figure 6d. As seen, CRP binding reached saturation after a 60 min incubation, so 60 min was selected as the optimal incubation time.

### 2.6. ECL Determination of CRP

**Figure 7:**
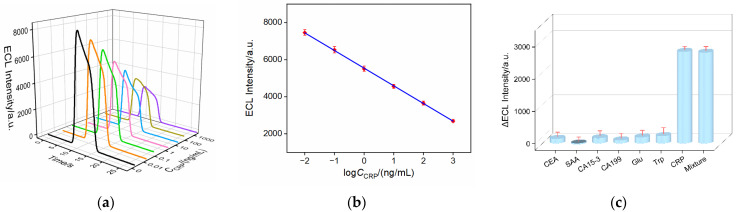
(**a**) ECL responses obtained when the aptasensor was incubated in the presence of different concentrations (0.01~1000 ng/mL) of CRP. (**b**) Corresponding calibration curve. Error bars represent the standard deviation of three measurements. The electrolyte was 0.01 M PBS (pH = 7.4) containing 3 mM TPA. The PMT voltage was 400 V. (**c**) The change of ECL intensity before and after incubation with CEA (1 ng/mL), SAA (1 ng/mL), CA15-3 (1 mU/mL), CA199 (1 mU/mL), Glu (100 μM), Trp (100 μM), CRP (1 ng/mL), or their mixture (1 ng/mL).

Figure 7a shows the ECL-time curves of the constructed aptasensor when incubated with different concentrations of CRP. It can be observed that the ECL intensity of the electrode gradually decreased with an increasing CRP concentration. The specific binding between the recognitive aptamer and CRP formed the aptamer–antigen complex, which reduced co-reactant diffusion. The higher the CRP concentration, the lower the ECL signal. The corresponding calibration curve in Figure 7b demonstrated a good linear relationship between the ECL intensity (I_ECL_) and the logarithm of the CRP concentration (logC_CRP_) in the range from 0.01 to 1000 ng/mL (I_ECL_ = −952.9 (±5.7) logC_CRP_ + 5544 (±14), R^2^ = 0.999), yielding a low detection limit (DL) of 8.5 pg/mL based on the signal/noise (S/N = 3). Thus, the developed sensor has the potential for the analysis of the CRP concentration in healthy individuals (≤1000 ng/mL) or patients (>1000 ng/mL, through dilution) [55]. Table 1 summarized the CRP detection performance using various sensors [56,57,58,59,60,61,62]. Compared to other sensors, the fabricated ECL aptasensor exhibited a wider linear detection range and lower DL. Compared to the multiple or complex nanomaterials that were required for other sensors, the SNF employed in this study did not require a complex preparation process, thus enhancing the convenience of sensor fabrication.

### 2.7. Selectivity and Stability of the Constructed Aptasensor

A series of biomarkers or metabolites present in biological samples were selected as potential interferents to assess the selectivity of the constructed ECL aptasensor. As shown in Figure 7c, the aptasensor was incubated with carcinoembryonic antigen (CEA), serum amyloid A (SAA), carbohydrate antigen 15-3 (CA15-3), carbohydrate antigen 19-9 (CA19-9), glucose (Glu), tryptophan (Trp), CRP, or their mixture, respectively. Only CRP or the mixture of these seven species led to a significant decrease in the ECL signal of the aptamer sensor. These results indicated that the sensor exhibited a high selectivity, attributed to the highly specific recognition between the aptamer and CRP. The aptamer sensor remained stable when stored at 4 °C for 11 days, with an ECL signal retained at 92.8% of the initial signal after incubation with CRP (1 ng/mL), demonstrating a good storage stability. In addition, five parallel aptasensors were fabricated, showing very similar response changes with an RSD of 2.0% for the determination of CRP (0.01 ng/mL), confirming a good reproducibility for the aptamer sensor.

### 2.8. Real Sample Analysis

To demonstrate the application of the constructed aptasensor for a real sample analysis, fetal bovine serum was employed as a model sample. The detection of CRP was investigated using the standard addition method. Different concentrations of CRP were added to the serum samples diluted 50-fold to simulate the different CRP levels in healthy individuals found in real samples. As shown in Table 2, the designed ECL aptamer sensor exhibited satisfactory detection recoveries ranging from 94.8% to 108% and a low RSD (<3.6%). This confirms the high accuracy of the aptasensor constructed in this study for detecting CRP in actual samples.

The stability of the bipolar layer during the storage period and contact with the spiked serum were investigated using SEM characterization. As revealed in Figure S1 in the supporting information (SI), the bipolar layer on the electrode, whether during storage or upon contact with serum, showed no collapse or rupture. The thickness remained consistent with that observed in Figure 2c, confirming the stability of the bipolar layer.

## 3. Materials and Methods

### 3.1. Chemicals and Materials

In this work, all the chemicals and reagents used were of analytical grade and were not further processed before use. The ultrapure water (18 MΩ·cm) used in the experiment was prepared using a Mill-Q system (Millipore Corporation, Boston, MA, USA). C-reactive protein (CRP) and serum amyloid A (SAA) were purchased from Okay Biotechnology Co., Ltd. (Nanjing, China). Carcinoembryonic antigen (CEA), carbohydrate antigen 15-3 (CA15-3), and carbohydrate antigen 19-9 (CA19-9) were obtained from KEY-BIO Biotech Co., Ltd. (Beijing, China). The CRP aptamer (5′-NH_2_-CGAAGGGGATTCGAGGGGTGATTGCGTGCTCCATTTGGTG-3′) [55] was obtained from Sangon Biotech Co., Ltd. (Shanghai, China). Sodium dihydrogen phosphate dihydrate (NaH_2_PO_4_·2H_2_O), disodium hydrogen phosphate dodecahydrate (Na_2_HPO_4_·12H_2_O), cetyltrimethylammonium bromide (CTAB), tetraethyl orthosilicate (TEOS, 98%), potassium hydrogen phthalate (KHP), potassium hexacyanoferrate (II) (K_3_[Fe(CN)_6_], 99.5%), tripropylamine (TPA), glutaraldehyde (GA), tryptophan (Trp), and glucose (Glu) were all purchased from Aladdin Bio-Chem Technology Co., Ltd. (Shanghai, China). Potassium hydrogen phthalate (KHP) and 3-aminopropyltriethoxysilane (APTES) were obtained from Macklin Reagent Co., Ltd. (Shanghai, China). Ruthenium (II) tris(bipyridine) chloride hexahydrate (Ru(bpy)_3_Cl_2_·6H_2_O) and bovine serum albumin (BSA) were purchased from Sigma-Aldrich (Shanghai, China). Anhydrous ethanol (99.8%) and concentrated hydrochloric acid (HCl, 38%) were acquired from Shuanglin Chemical Reagent Co., Ltd. (Hangzhou, China). Sodium nitrate (NaNO_3_), potassium chloride (KCl), and sodium hydroxide (NaOH) were obtained from High Jintai Fine Chemical Co., Ltd. (Hangzhou, China). Phosphate-buffered saline (PBS) was prepared by mixing NaH_2_PO_4_ and Na_2_HPO_4_ in the appropriate proportions. The supporting electrode, indium tin oxide (ITO) conductive glass electrodes with a sheet resistance lower than 17 Ω/square and thickness of 100 ± 20 nm were purchased from Kaiwei Optoelectronic Technology Co., Ltd. (Zhuhai, China).

### 3.2. Characteriaztions and Instrumentations

The morphology and thickness of bp-SNF/ITO were characterized using scanning electron microscopy (SEM, Sigma 300, ZEISS, Baden-Württemberg, Germany) and transmission electron microscopy (TEM, HT7700, Hitachi, Kyoto, Japan). For the preparation of SEM samples, the non-conductive side of bp-SNF/ITO was gently scratched with a glass cutter to create marks and then the electrode was broken to obtain a neat cross-section. Subsequently, the samples were gold-coated and observed in the SEM sample chamber with an acceleration voltage set at 5 kV. For the TEM sample preparation, bp-SNF was gently scraped from the ITO electrode surface into a 1.5 mL centrifuge tube using a scalpel. The, the scraped sample was dispersed in a 200 µL ethanol solution followed by ultrasonic dispersion. The resulting supernatant was dropped onto a copper grid, air-dried, and subsequently observed under TEM with an acceleration voltage of 200 kV.

All electrochemical tests, including cyclic voltammetry (CV) and differential pulse voltammetry (DPV), were conducted on the CHI660E electrochemical workstation from Shanghai Chenhua Instrument Co., Ltd. (Shanghai, China). ECL tests were performed using a self-constructed ECL analysis system. The photomultiplier tube (PMT) was set to a voltage of 400 V. Both ECL and EC tests employed a three-electrode system, with bare or modified ITO electrode as the working electrode, an Ag/AgCl electrode (saturated with KCl) as the reference electrode. A platinum wire electrode served as the counter electrode for EC measurements, while a platinum sheet (2 cm × 4 cm) was used as the counter electrode for the ECL measurements.

### 3.3. Preparation of n-SNF/ITO Electrode

The n-SNF was modified on the ITO electrode (2.5 cm × 5 cm) using the Stöber solution growth method [63]. Before use, it is necessary to ultrasonically clean the ITO glass in a 1 M NaOH aqueous solution for 1 h, followed by separate ultrasonication in acetone and ethanol for 30 min each. Finally, the ITO electrode was rinsed three times with ultrapure water using ultrasonication. For the growth of n-SNF, a mixture containing tetraethyl orthosilicate (TEOS) precursor and cetyltrimethylammonium bromide (CTAB) surfactant micelles (SM) was prepared. Specifically, 160 mg of CTAB was added in an ethanol-water solution (*V*_ethanol_:*V*_water_ = 3:7, 100 mL) under stirring for 5 min for complete dissolving. Then, ammonia solution (10%, 100 μL) and TEOS (80 μL) were sequentially added under stirring, until the solution became clear and bubble-free. Next, the clean ITO electrode was placed in the solution and the reaction was performed in a 60 °C water bath for 24 h. After the reaction was completed, the electrode was rinsed with a large amount of ultrapure water, and then aged overnight at 100 °C to obtain a modified electrode with SM templates, named SM@n-SNF/ITO. The SM@n-SNF/ITO was then immersed in a 0.1 M HCl-ethanol solution and stirred for 5 min to remove the micelles, resulting in an open-channel n-SNF/ITO electrode.

### 3.4. Preparation of bp-SNF/ITO and Ru@bp-SNF/ITO Electrode

p-SNF was grown on the surface of n-SNF/ITO using the electrochemical-assisted self-assembly (EASA) method [64,65]. Firstly, a precursor solution containing mixed siloxanes of TEOS and APTES, along with CTAB micellar templates, was prepared. Specifically, in a mixed solution containing 20 mL of ethanol and 20 mL of NaNO_3_ (0.1 M, pH = 2.6), 1.585 g of CTAB as the template agent and 318 μL of APTES were added. After stirring to dissolve, the pH was adjusted to 2.97, and then 2372 μL of TEOS was added, followed by stirring at room temperature for 2.5 h. When the n-SNF/ITO electrode was placed in the precursor solution, a constant current (−0.7 mA/cm^2^) was applied for 15 s. After the reaction, the electrode was quickly removed and rinsed with a large amount of ultrapure water, and aged overnight at 120 °C to obtain the SM@bp-SNF/ITO electrode. Subsequently, the electrode was stirred in a 0.1 M HCl-ethanol solution for 5 min to remove the micelles, resulting in the bipolar-film-modified electrode (bp-SNF/ITO).

To introduce the Ru(bpy)_3_^2+^ probe into the nanochannels of bp-SNF, the bp-SNF/ITO electrode was immersed in a PBS solution (0.01 M, pH = 7.4) containing 1 mM of Ru(bpy)_3_^2+^ and stirred for 20 min. After rinsing with ultrapure water, the electrode fixed with Ru(bpy)_3_^2+^ was obtained, denoted as Ru@bp-SNF/ITO. As a control, Ru(bpy)_3_^2+^ was enriched on an n-SNF/ITO electrode using the same procedure. The resulting electrode was referred to as Ru@n-SNF/ITO.

### 3.5. Fabrication of Aptasensor

To construct the recognition interface for CRP determination, the corresponding aptamer (Apt) was covalently immobilized on the outer surface of bp-SNF. Firstly, the SM@bp-SNF/ITO electrode was immersed in a 5% GA solution (0.01 M PBS, pH 7.4) and reacted at 37 °C for 20 min in the dark to obtain an aldehyde-functionalized surface. Afterwards, the removal of micelles led to the formation of the aldehyde-derived electrode (GA/Ru@bp-SNF/ITO electrode). Then, the GA/Ru@bp-SNF/ITO electrode was immersed in an Apt solution (0.3 μM) and incubated at 4 °C for 1 h. This was followed by incubation at room temperature for 30 min in a BSA solution (0.5 wt% in 0.01 M PBS, pH 7.4) to block non-specific binding sites, resulting in the aptamer sensor, designated as Apt/GA/Ru@bp-SNF/ITO.

### 3.6. ECL Determination of CRP

CRP was detected using the Apt/GA/Ru@bp-SNF/ITO electrode. The constructed aptamer sensor was incubated with different concentrations of CRP at 4 °C for 1 h, followed by washing with PBS (0.01 M, pH 7.4). The ECL signals of the electrode before and after CRP binding were determined. Specifically, the electrode was immersed in a solution containing co-reactant and tripropylamine (TPA, 3 mM in 0.01 M PBS, pH 7.4), and continuous cyclic scanning between 0 and 1.4 V was conducted at a scan rate of 100 mV/s to trigger the ECL process. The ECL signals and corresponding CV curves were simultaneously recorded at room temperature. Using fetal bovine serum as a model, the analytical performance of the constructed aptamer sensor for a real sample analysis was evaluated using the standard addition method. Prior to detection, the serum samples were diluted 50-fold with PBS (0.01 M, pH 7.4).

## 4. Conclusions

In summary, a novel ECL aptasensor was fabricated based on bipolar nanochannel array film (bp-SNF). This allowed for the efficient confinement of the electrochemical luminescent species, Ru(bpy)_3_^2+^, resulting in a significant enhancement of the ECL signal. The dual-layer structure of bp-SNF, with its charge-reversal properties, facilitated dual electrostatic interactions with Ru(bpy)_3_^2+^, contributing to the stable confinement of the probe.

Recognition aptamers for CRP were covalently immobilized on the outer surface of bp-SNF. Through the development of this probe-immobilized aptasensor, the ECL determination of CRP was achieved with a high sensitivity. Compared to previously published sensors, the proposed ECL aptasensor exhibited a relatively wide linear range and low LOD for the determination of CRP, without the need for synthesizing complex nanomaterials. Compared to the traditional immunoassay, such as enzyme-linked immunosorbent assay (ELISA) based on forming a sandwich-type immunocomplex, the developed aptasensor utilizes aptamers as recognition ligands. The immobilization process of aptamers is similar to the immobilization step of antibodies in traditional immunosensors. However, DNA-based aptamers are cost-effective and demonstrate a higher stability than protein-based antibodies. The fabricated aptasensor exhibits the advantages of cost effectiveness and the accessibility of the supporting electrodes, the convenience and low cost of bp-SNF fabrication, and a high probe immobilization capacity and stability, offering significant applications for rapid and highly sensitive biomarker detection.

## Figures and Tables

**Table 1:** Comparison of CRP detection performance using different sensors.

Materials	Sensor	Linear Range (ng/mL)	LOD (pg/mL)	Incubation Time (min)	Probe Mode	Ref.
TiNTs/PtNWs/ITO	ECL immunosensor	0.05–6.25	11	60	Free	[56]
Ru(bpy)_3_^2+^-labeled AuNPs	ECL immunosensor	0.01–1000	4.6	60	Immobilized	[57]
JNP/CRP/Apt-AuNPs/SPCE	AMP aptasensor	0.01–1	3.1	30	Free	[58]
PMPC-SH/AuNPs-SPCE	EC sensor	5–5000	0.00155	60	Free	[59]
L-cys/AuNPs/G/SPCE	EC immunosensor	50–10^5^	15000	50	Free	[60]
MF-DNA-4WJ/pRhNPs-	EC aptasensor	0.23–23,000	80	90	Free	[61]
GBP/AuNPs@BP@PDA	EC biosensor	0–36	700	120	Free	[62]
Ru@bp-SNA/ITO	ECL aptasensor	0.01–1000	8.5	60	Immobilized	This work

TiNTs, titania nanotubes; PtNWs, platinum nanowire; AuNPs, gold nanoparticles; JNP, Janus nanoparticles; SPCE, screen-printed carbon electrodes; AMP, amperometry; EC, electrochemical; PMPC-SH, thiol-terminated poly (2-methacryloyloxyethylphosphorylcholine); L-cys, L-Cysteine; G, graphene; MF-DNA-4WJ, multifunctional DNA four-way junction; pRhNPs, porous rhodium nanoparticle; GBP, gold binding peptide; and AuNPs@BP@PDA, gold nanoparticles tethered polydopamine—black phosphorus.

**Table 2:** Detection of CRP in fetal bovine serum with the aptasensor.

Sample	Added (ng/mL)	Found (ng/mL)	Recovery (%)	RSD (%, *n* = 3)
Serum *	0.100	0.0974 ± 0.0018	97.4	1.8
	10.0	10.8 ± 0.035	108	3.2
	100	94.8 ± 3.4	94.8	3.6

* The serum samples were diluted 50 times with PBS (0.01 M, pH 7.4).

## Data Availability

The data presented in this study are available on request from the corresponding author.

## Supplementary Materials

The following supporting information can be downloaded at: https://www.mdpi.com/article/10.3390/molecules28237867/s1, Figure S1: Cross-sectional SEM image of bp-SNF/ITO that have been stored for 11 days (a) or after contacted with the spiked serum (b).

## References

[1] Cui F., Zhou Z., Zhou H.S. (2020). Review—Measurement and analysis of cancer biomarkers based on electrochemical biosensors. J. Electrochem. Soc..

[2] Li Y., Zhong X., Cheng G., Zhao C., Zhang L., Hong Y., Wan Q., He R., Wang Z. (2017). Hs-CRP and all-cause, cardiovascular, and cancer mortality risk: A meta-analysis. Atherosclerosis.

[3] Shamsuzzaman A.S.M., Winnicki M., Lanfranchi P., Wolk R., Kara T., Accurso V., Somers V.K. (2002). Elevated C-reactive protein in patients with obstructive sleep apnea. Circulation.

[4] Danesh J., Wheeler J.G., Hirschfield G.M., Eda S., Eiriksdotti G., Rumley A., Lowe G.D.O., Pepys M.B., Gudnason V. (2004). C-reactive protein and other circulating markers of inflammation in the prediction of coronary heart disease. N. Engl. J. Med..

[5] Mazer S.P., Rabbani L.E. (2004). Evidence for C-reactive protein’s role in (crp) vascular disease: Atherothrombosis, immuno-regulation and CRP. J. Thromb. Thrombolys..

[6] Fleischmann R.M., van der Heijde D., Gardiner P.V., Szumski A., Marshall L., Bananis E. (2017). DAS28-CRP and DAS28-ESR cut-offs for high disease activity in rheumatoid arthritis are not interchangeable. RMD Open.

[7] Pearson T.A., Mensah G.A., Alexander R.W., Anderson J.L., Cannon R.O., Criqui M., Fadl Y.Y., Fortmann S.P., Hong Y., Myers G.L. (2003). Markers of inflammation and cardiovascular disease. Circulation.

[8] Lee M.-H., Liu K.-H., Thomas J.L., Chen C.-Y., Chen C.-Y., Yang C.-H., Lin H.-Y. (2022). Doping of MXenes enhances the electrochemical response of peptide-imprinted conductive polymers for the recognition of C-Reactive protein. Biosens. Bioelectron..

[9] Chandra P. (2014). Prospects and advancements in C-reactive protein detection. World J. Methodol..

[10] Sproston N.R., Ashworth J.J. (2018). Role of C-Reactive Protein at Sites of Inflammation and Infection. Front. Immunol..

[11] Thompson D., Pepys M.B., Wood S.P. (1999). The physiological structure of human C-reactive protein and its complex with phosphocholine. Structure.

[12] Ma N., Luo X., Wu W., Liu J. (2022). Fabrication of a disposable electrochemical immunosensor based on nanochannel array modified electrodes and gated electrochemical signals for sensitive determination of C-reactive protein. Nanomaterials.

[13] Dhiman A., Kalra P., Bansal V., Bruno J.G., Sharma T.K. (2017). Aptamer-based point-of-care diagnostic platforms. Sens. Actuators B Chem..

[14] Chen X., Liu X., Zhang C., Meng H., Liu B., Wei X. (2022). A rapid fluorescent aptasensor for point-of-care detection of C-reactive protein. Talanta.

[15] Yan Z., Zhang S., Liu J., Xing J. (2023). Homogeneous electrochemical aptamer sensor based on two-dimensional nanocomposite probe and nanochannel modified electrode for sensitive detection of carcinoembryonic antigen. Molecules.

[16] Zhang T., Yang L., Yan F., Wang K. (2023). Vertically-ordered mesoporous silica film based electrochemical aptasensor for highly sensitive detection of alpha-fetoprotein in human serum. Biosensors.

[17] Ilgu M., Nilsen-Hamilton M. (2016). Aptamers in analytics. Analyst.

[18] Sarkar D.J., Behera B.K., Parida P.K., Aralappanavar V.K., Mondal S., Dei J., Das B.K., Mukherjee S., Pal S., Weerathunge P. (2023). Aptamer-based NanoBioSensors for seafood safety. Biosens. Bioelectron..

[19] Hicke B.J., Stephens A.W. (2000). Escort aptamers: A delivery service for diagnosis and therapy. J. Clin. Investig..

[20] Cesewski E., Johnson B.N. (2020). Electrochemical biosensors for pathogen detection. Biosens. Bioelectron..

[21] Kazemi S.H., Ghodsi E., Abdollahi S., Nadri S. (2016). Porous graphene oxide nanostructure as an excellent scaffold for label-free electrochemical biosensor: Detection of cardiac troponin I. Mater. Sci. Eng. C.

[22] Zhang T., Xu S., Lin X., Liu J., Wang K. (2023). Label-free electrochemical aptasensor based on the vertically-aligned mesoporous silica films for determination of aflatoxin B1. Biosensors.

[23] Shi Z., Li G., Hu Y. (2019). Progress on the application of electrochemiluminescence biosensor based on nanomaterials. Chin. Chem. Lett..

[24] Huang J., Zhang T., Zheng Y., Liu J. (2023). Dual-mode sensing platform for cancer antigen 15-3 determination based on a silica nanochannel array using electrochemiluminescence and electrochemistry. Biosensors.

[25] Luo X., Zhang T., Tang H., Liu J. (2022). Novel electrochemical and electrochemiluminescence dual-modality sensing platform for sensitive determination of antimicrobial peptides based on probe encapsulated liposome and nanochannel array electrode. Front. Nutr..

[26] Zheng Y., Lin J., Xie L., Tang H., Wang K., Liu J. (2021). One-step preparation of nitrogen-doped graphene quantum dots with anodic electrochemiluminescence for sensitive detection of hydrogen peroxide and glucose. Front. Chem..

[27] Gong J., Zhang T., Luo T., Luo X., Yan F., Tang W., Liu J. (2022). Bipolar silica nanochannel array confined electrochemiluminescence for ultrasensitive detection of SARS-CoV-2 antibody. Biosens. Bioelectron..

[28] Ma K., Zheng Y., An L., Liu J. (2022). Ultrasensitive immunosensor for prostate-specific antigen based on enhanced electrochemiluminescence by vertically ordered mesoporous silica-nanochannel film. Front. Chem..

[29] Chen H., Huang J., Zhang R., Yan F. (2022). Dual-mode electrochemiluminescence and electrochemical sensor for alpha-fetoprotein detection in human serum based on vertically ordered mesoporous silica films. Front. Chem..

[30] Wang S., Luo J., He Y., Chai Y., Yuan R., Yang X. (2018). Combining porous magnetic Ni@C nanospheres and CaCO_3_ microcapsule as surface-enhanced raman spectroscopy sensing platform for hypersensitive C-reactive protein detection. ACS Appl. Mater. Interfaces.

[31] Zhang C., Zhou X., Yan F., Lin J. (2023). N-doped graphene quantum dots confined within silica nanochannels for enhanced electrochemical detection of doxorubicin. Molecules.

[32] Beiranvand B., Khabibullin R.A., Sobolev A.S. (2023). Local Field Enhancement Due to the Edge States of Nanoplasmonic Crystal. Photonics.

[33] Shi X., Fan X., Zhu Y., Liu Y., Wu P., Jiang R., Wu B., Wu H.-A., Zheng H., Wang J. (2022). Pushing detectability and sensitivity for subtle force to new limits with shrinkable nanochannel structured aerogel. Nat. Commun..

[34] Zhao J., Duan W., Liu X., Xi F., Wu J. (2023). Microneedle patch integrated with porous silicon confined dual nanozymes for synergistic and hyperthermia-enhanced nanocatalytic ferroptosis treatment of melanoma. Adv. Funct. Mater..

[35] He J., Li Z., Zhao R., Lu Y., Shi L., Liu J., Dong X., Xi F. (2019). Aqueous synthesis of amphiphilic graphene quantum dots and their application as surfactants for preparing of fluorescent polymer microspheres. Colloid Surface A.

[36] Xu S., Zhang S., Li Y., Liu J. (2023). Facile synthesis of iron and nitrogen Co-doped carbon dot nanozyme as highly efficient peroxidase mimics for visualized detection of metabolites. Molecules.

[37] Lin J., Li K., Wang M., Chen X., Liu J., Tang H. (2020). Reagentless and sensitive determination of carcinoembryonic antigen based on a stable Prussian blue modified electrode. RSC Adv..

[38] Zhang J., Yang L., Pei J., Tian Y., Liu J. (2022). A reagentless electrochemical immunosensor for sensitive detection of carcinoembryonic antigen based on the interface with redox probe-modified electron transfer wires and effectively immobilized antibody. Front. Chem..

[39] Walcarius A. (2021). Electroinduced surfactant self-assembly driven to vertical growth of oriented mesoporous films. Acc. Chem. Res..

[40] Zhou P., Yao L., Chen K., Su B. (2019). Silica nanochannel membranes for electrochemical analysis and molecular sieving: A comprehensive review. Crit. Rev. Anal. Chem..

[41] Han Q., Zhang T., Wang M., Yan F., Liu J. (2022). Disposable electrochemical sensors for highly sensitive detection of chlorpromazine in human whole blood based on the silica nanochannel array modified screen-printed carbon electrode. Molecules.

[42] Zhou X., Han Q., Zhou J., Liu C., Liu J. (2023). Reagentless electrochemical detection of tumor biomarker based on stable confinement of electrochemical probe in bipolar silica nanochannel film. Nanomaterials.

[43] Yang L., Zhang T., Zhou H., Yan F., Liu Y. (2022). Silica nanochannels boosting Ru(bpy)_3_^2+^-mediated electrochemical sensor for the detection of guanine in beer and pharmaceutical samples. Front. Nutr..

[44] Zheng W., Su R., Yu G., Liu L., Yan F. (2022). Highly sensitive electrochemical detection of paraquat in environmental water samples using a vertically ordered mesoporous silica film and a nanocarbon composite. Nanomaterials.

[45] Yan F., Chen J., Jin Q., Zhou H., Sailjoi A., Liu J., Tang W. (2020). Fast one-step fabrication of a vertically-ordered mesoporous silica-nanochannel film on graphene for direct and sensitive detection of doxorubicin in human whole blood. J. Mater. Chem. C.

[46] Yan F., Luo T., Jin Q., Zhou H., Sailjoi A., Dong G., Liu J., Tang W. (2021). Tailoring molecular permeability of vertically-ordered mesoporous silica-nanochannel films on graphene for selectively enhanced determination of dihydroxybenzene isomers in environmental water samples. J. Hazard. Mater..

[47] Wang K., Yang L., Huang H., Lv N., Liu J., Liu Y. (2022). Nanochannel array on electrochemically polarized screen printed carbon electrode for rapid and sensitive electrochemical determination of clozapine in human whole blood. Molecules.

[48] Ma X., Liao W., Zhou H., Tong Y., Yan F., Tang H., Liu J. (2020). Highly sensitive detection of rutin in pharmaceuticals and human serum using ITO electrodes modified with vertically-ordered mesoporous silica-graphene nanocomposite films. J. Mater. Chem. B.

[49] Cui Y., Zhang S., Zhou X., Yan F., Hu W. (2023). Silica nanochannel array on co-electrodeposited graphene-carbon nanotubes 3D composite film for antifouling detection of uric acid in human serum and urine samples. Microchem. J..

[50] Zhou H., Ma X., Sailjoi A., Zou Y., Lin X., Yan F., Su B., Liu J. (2022). Vertical silica nanochannels supported by nanocarbon composite for simultaneous detection of serotonin and melatonin in biological fluids. Sens. Actuators B Chem..

[51] Deng X., Lin X., Zhou H., Liu J., Tang H. (2023). Equipment of vertically-ordered mesoporous silica film on electrochemically pretreated three-dimensional graphene electrodes for sensitive detection of methidazine in urine. Nanomaterials.

[52] Wang M., Lin J., Gong J., Ma M., Tang H., Liu J., Yan F. (2021). Rapid and sensitive determination of doxorubicin in human whole blood by vertically-ordered mesoporous silica film modified electrochemically pretreated glassy carbon electrodes. RSC Adv..

[53] Ma K., Yang L., Liu J., Liu J. (2022). Electrochemical sensor nanoarchitectonics for sensitive detection of uric acid in human whole blood based on screen-printed carbon electrode equipped with vertically-ordered mesoporous silica-nanochannel film. Nanomaterials.

[54] Li D., Xu S., Jin H., Wang J., Yan F. (2023). Copper nanoparticles confined in a silica nanochannel film for the electrochemical detection of nitrate ions in water samples. Molecules.

[55] Huang S., Liu Z., Yan Y., Chen J., Yang R., Huang Q., Jin M., Shui L. (2022). Triple signal-enhancing electrochemical aptasensor based on rhomboid dodecahedra carbonized-ZIF67 for ultrasensitive CRP detection. Biosens. Bioelectron..

[56] Rong Z., Chen F., Jilin Y., Yifeng T. (2019). A C-reactive protein immunosensor based on platinum nanowire/titania nanotube composite sensitized electrochemiluminescence. Talanta.

[57] Hong D., Kim K., Jo E.-J., Kim M.-G. (2021). Electrochemiluminescence-incorporated lateral flow immunosensors using Ru(bpy)_3_^2+^-labeled gold nanoparticles for the full-range detection of physiological C-reactive protein levels. Anal. Chem..

[58] Villalonga A., Sánchez A., Vilela D., Mayol B., Martínez-Ruíz P., Villalonga R. (2022). Electrochemical aptasensor based on anisotropically modified (Janus-type) gold nanoparticles for determination of C-reactive protein. Microchim. Acta.

[59] Pinyorospathum C., Chaiyo S., Sae-ung P., Hoven V.P., Damsongsang P., Siangproh W., Chailapakul O. (2019). Disposable paper-based electrochemical sensor using thiol-terminated poly(2-methacryloyloxyethyl phosphorylcholine) for the label-free detection of C-reactive protein. Microchim. Acta.

[60] Boonkaew S., Chaiyo S., Jampasa S., Rengpipat S., Siangproh W., Chailapakul O. (2019). An origami paper-based electrochemical immunoassay for the C-reactive protein using a screen-printed carbon electrode modified with graphene and gold nanoparticles. Microchim. Acta.

[61] Kim J., Park J.-A., Yim G., Jang H., Kim T.-H., Sohn H., Lee T. (2021). Fabrication of an electrochemical biosensor composed of multi-functional Ag ion intercalated DNA four-way junctions/rhodium nanoplate heterolayer on a micro-gap for C-reactive protein detection in human serum. Analyst.

[62] Yang H.J., Kim M.W., Raju C.V., Cho C.H., Park T.J., Park J.P. (2023). Highly sensitive and label-free electrochemical detection of C-reactive protein on a peptide receptor−gold nanoparticle−black phosphorous nanocomposite modified electrode. Biosens. Bioelectron..

[63] Teng Z., Zheng G., Dou Y., Li W., Mou C.-Y., Zhang X., Asiri A.M., Zhao D. (2012). Highly ordered mesoporous silica films with perpendicular mesochannels by a simple Stöber-solution growth approach. Angew. Chem. Int. Ed..

[64] Walcarius A., Sibottier E., Etienne M., Ghanbaja J. (2007). Electrochemically assisted self-assembly of mesoporous silica thin films. Nat. Mater..

[65] Gong J., Zhang T., Chen P., Yan F., Liu J. (2022). Bipolar silica nanochannel array for dual-mode electrochemiluminescence and electrochemical immunosensing platform. Sens. Actuators B Chem..

