# Supplementary material for: The Fabrication of a Probe-Integrated Electrochemiluminescence Aptasensor Based on Double-Layered Nanochannel Array with Opposite Charges for the Sensitive Determination of C-Reactive Protein
Source: Molecules. 2023 Nov 30;28(23):7867. doi: 10.3390/molecules28237867 (PMC10708393; doi:10.3390/molecules28237867)
Supplement: Supplementary file 1 [file molecules-28-07867-s001.zip › molecules-2675716-supplementary.pdf]

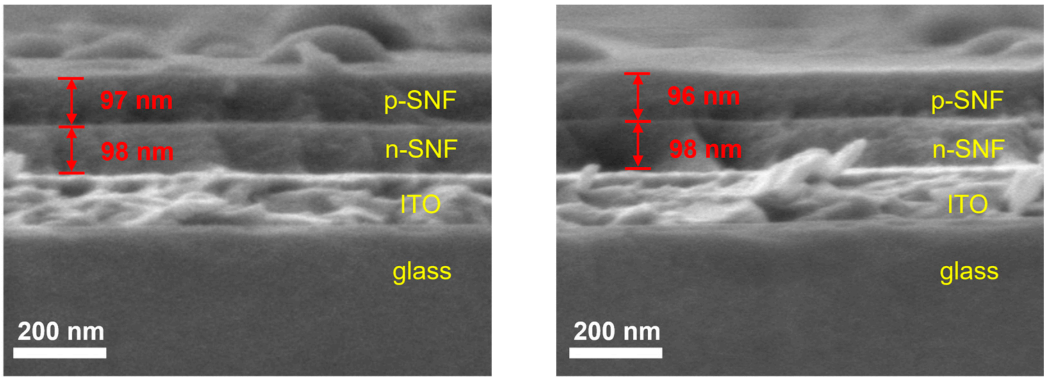

(**a**) (**b**)

Figure S1. Cross-sectional SEM image of bp-SNF/ITO that have been stored for 11 days (**a**) or after contacted with the spiked serum (**b**).
